# Supplementary material for: Brain-wide functional connectome analysis of 40,000 individuals reveals brain networks that show aging effects in older adults
Source: Imaging Neurosci (Camb). 2024 Dec 16;2:imag-2-00394. doi: 10.1162/imag_a_00394 (PMC12315726; doi:10.1162/imag_a_00394)
Supplement: Supplementary Material [file imag_a_00394-supp.pdf]

SUPPLEMENTARY METHODS 2

SI.1 rfMRI data preprocessing 2

SI.1.1 rfMRI data acquisition 2

SI.1.2 rfMRI data preprocessing 2

SI.1.3 UKB rfMRI data quality control 2

SI.1.4 HCP-A rfMRI data quality control 2

Supplementary Figure. Measures of variation in the rsFC extracted from HCP-A rfMRI data. 3

SUPPLEMENTARY RESULTS 4

Figure S1. Sex subgroup analysis. 4

Figure S2. Sensitivity analysis. 4

Figure S3. 3-D demonstration of extracted subnetworks from UKB and HCP-A. 5

Figure S4. Mediation effect of structural changes on the age-related functional declines. 6

SI.2 Age-related subnetworks by different brain atlases 7

SI.2.1 Schaefer200 7

SI.2.2 Glasser360 7

SI.2.3 Power264 7

Figure S5. Whole-brain functional connectome derived from various brain atlases, showing age-related differences. 8

Figure S6. Age-related subnetworks derived from various brain atlases. 9

REFERENCE 10

Supplementary Methods

SI.1 rfMRI data preprocessing

SI.1.1 rfMRI data acquisition

In UKB, the MRI scans were performed on Siemens Skyra 3-Tesla with a Siemens 32-channel RF receive head coil (protocols available at https://biobank.ctsu.ox.ac.uk/crystal/crystal/docs/brain_mri.pdf). rfMRI data were obtained during a 6min session (490 timepoints, repetition time = 735 ms, echo time = 39 ms, multi-band acceleration 8) with a 2.4mm isotropic resolution and 64 slices.

In HCP-A, the MRI scans were performed on Siemens Prisma 3-Tesla with a Siemens 32-channel Prisma head coil (protocols available at https://www.humanconnectome.org/study/hcp-lifespan-aging/project-protocol/imaging-protocols-hcp-aging). rfMRI data were obtained during a 6min41s-long session (488 timepoints, repetition time = 800 ms, echo time = 37 ms, multi-band acceleration 8) with a 2.0mm isotropic resolution and 72 slices.

SI.1.2 rfMRI data preprocessing

Downloaded rfMRI data from UKB underwent echo-planar imaging (EPI) unwarping, gradient distortion correction (GDC), and motion correction using MC-FLIRT (Jenkinson et al., 2002) to reduce interpolation artefacts, and was then FIX-cleaned (Salimi-Khorshidi et al., 2014) to remove structural artefacts.

Downloaded rfMRI data from HCP-A underwent volumetric preprocessing (including GDC, motion correction using FLIRT, slice timing correction, spatial normalization, and spatial smoothing) and surface-based preprocessing (including surface reconstruction, registration to the structural T1w image, and smoothing) (Glasser et al., 2013; Smith et al., 2013). Additionally, multi-run FIX (MR-FIX) was applied to yield better separation of signal and noise components.

SI.1.3 UKB rfMRI data quality control

All UKB imaging data was subject to automated quality control based on a standard preprocessing pipeline (Alfaro-Almagro et al., 2018), in which subjects were excluded if their T1-weighted structural scans failed to map to standard space due to missingness, bad head movement, bad field/contrast problem, atypical structure, etc. The full list of quality control measures can be found elsewhere (Alfaro-Almagro et al., 2018).

SI.1.4 HCP-A rfMRI data quality control

All HCP-A subjects were subject to quality control measures at various stages, starting with real-time oversight during data acquisition and followed by manual and automated image reviews after acquisition.

We conducted additional quality control measures on the preprocessed HCP-A rfMRI data in response to observed instances of missing data and signal drops in the rfMRI time series. Our approach involved two main steps:

1) We initially excluded all participants whose rsFC data had a missing rate exceeding 5%.

2) To address unexpected signal drops that resulted in extremely high temporal correlations and minimal rsFC variations, we derived a measure to identify participants meeting either of the following criteria:

    a. The ratio of rsFC values larger than 0.9 over all rsFC edges > 0.1 (Figure A below).
    b. The standard deviation of rsFC values < 0.1 (Figure B below).

As a result of these quality control procedures, a total of 226 participants (30%) were excluded from the HCP-A cohort.

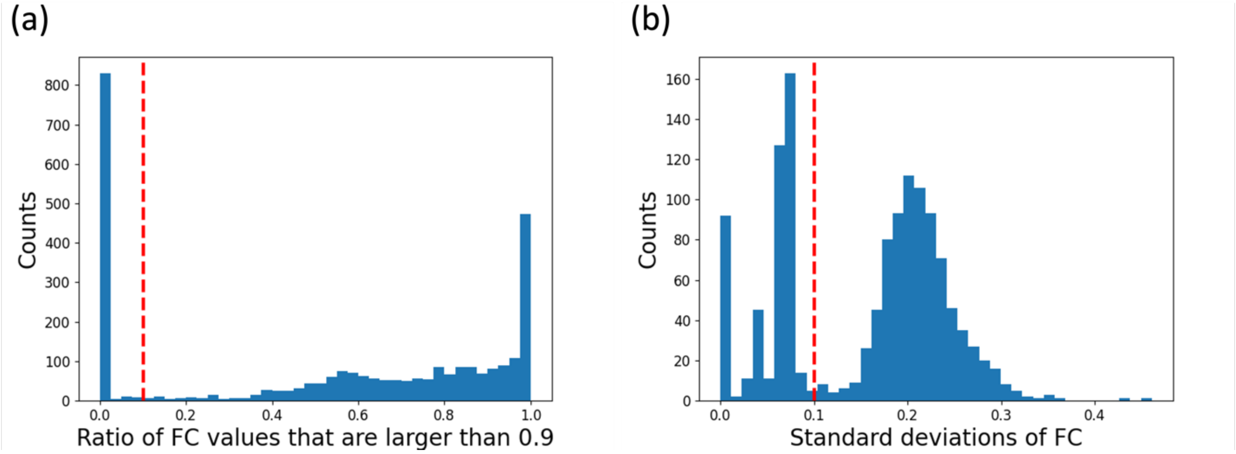

Supplementary Figure. Measures of variation in the rsFC extracted from HCP-A rfMRI data. Panel (a) shows a histogram illustrating the ratio of rsFC values exceeding 0.9 across all rsFC edges. The red dashed line at 0.1 represents the threshold we implemented to prevent rsFC with extremely large values from dominating the dataset, which typically occurs because the time series suffer from signal drops due to over-smoothing. Such signal drops in turn can cause the time series to have a correlation (i.e., rsFC) close to 1. This threshold was applied to ensure rsFC sufficient variation. Panel (b) shows the standard deviation of the rsFC data. The red dashed line at 0.1 signifies the cutoff we used to further ensure variation in the rsFC data.

Supplementary Results

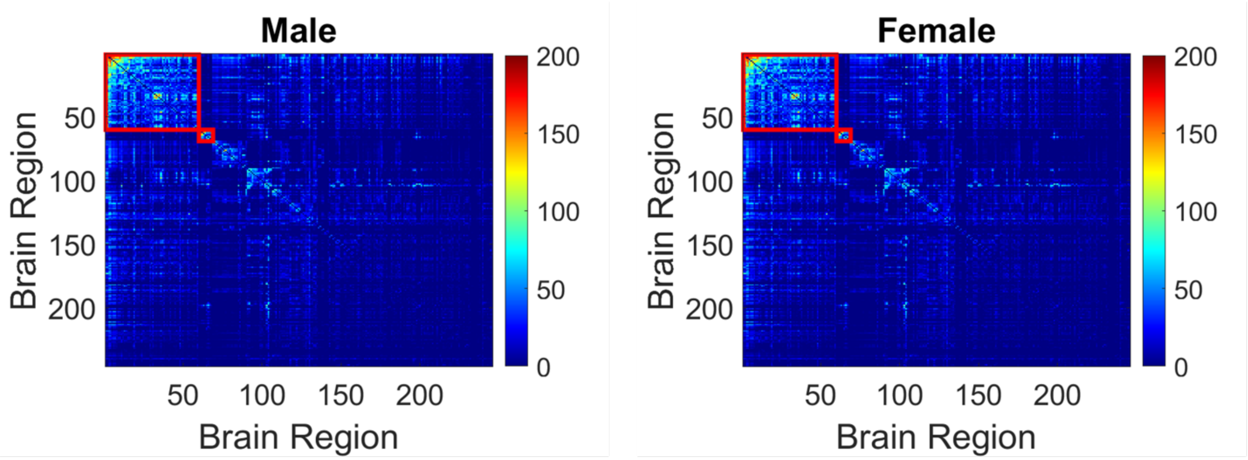

Figure S1. Sex subgroup analysis. We performed sex-specific subgroup analysis to assess if there’s any difference in age-related functional change patterns between sex. The figures show the subnetworks extracted using the data-driven subnetwork detection method, highlighted within red squares. Each matrix element is the -log10(p-values) obtained from association analysis between each rsFC and age across all subjects within the specific sex group. No difference was observed between the two groups.

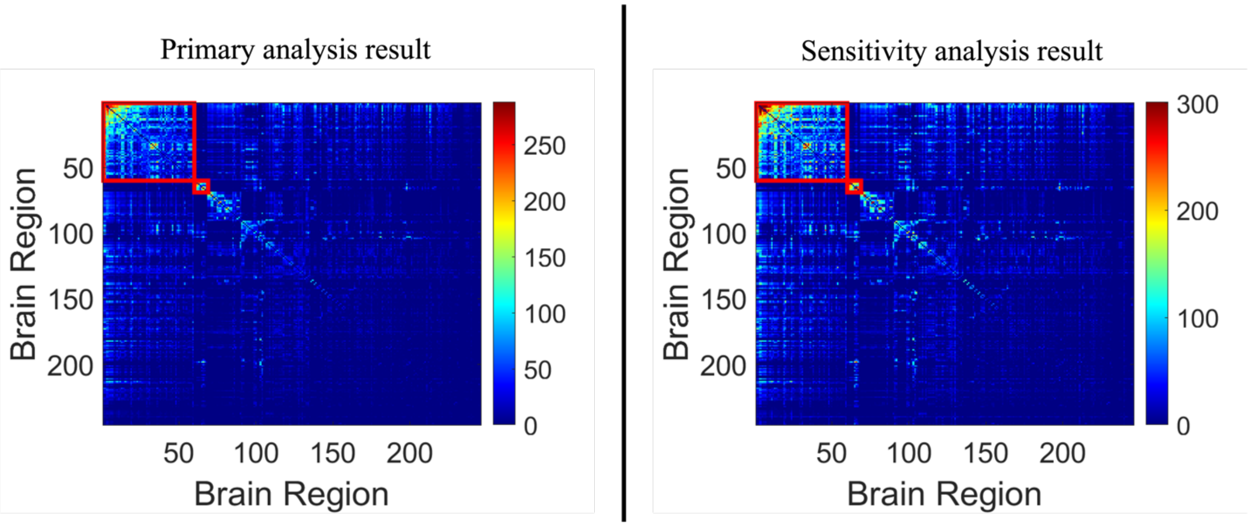

Figure S2. Sensitivity analysis. We performed sensitivity analysis on the entire UKB cohort that passed imaging quality check. The sensitivity cohort included 1,248 more subjects than our primary analysis cohort- these were the subjects originally excluded due to their health conditions such as neurological diseases or brain injuries. The highlighted red boxes represent the extracted age-related subnetworks, with each element denoting the -log10(p-values) obtained from association analysis between each

rsFC and age across all subjects. The age-related signals become stronger due to a larger sample size, but the difference in the subnetwork patterns is negligible.

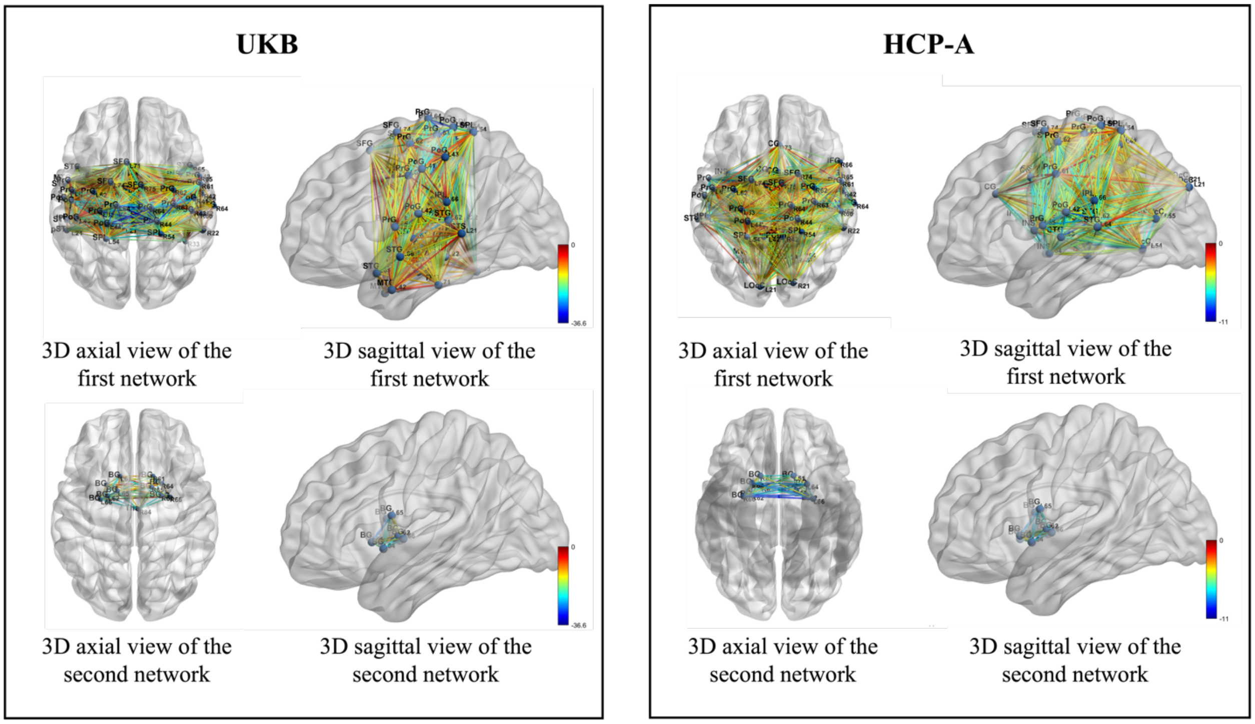

Figure S3. 3-D demonstration of extracted subnetworks from UKB and HCP-A. The left panel displays the axial and sagittal views of the first and second age-related subnetworks in UKB, while the right panel presents the corresponding 3-D visualization of age-related subnetworks in HCP-A. Connection colors denote the t-statistics derived from the association analysis between each rsFC and age, with the blue color signifying a larger effect size. Given our focus on age-related rsFC declines, positive associations are not depicted in this representation.

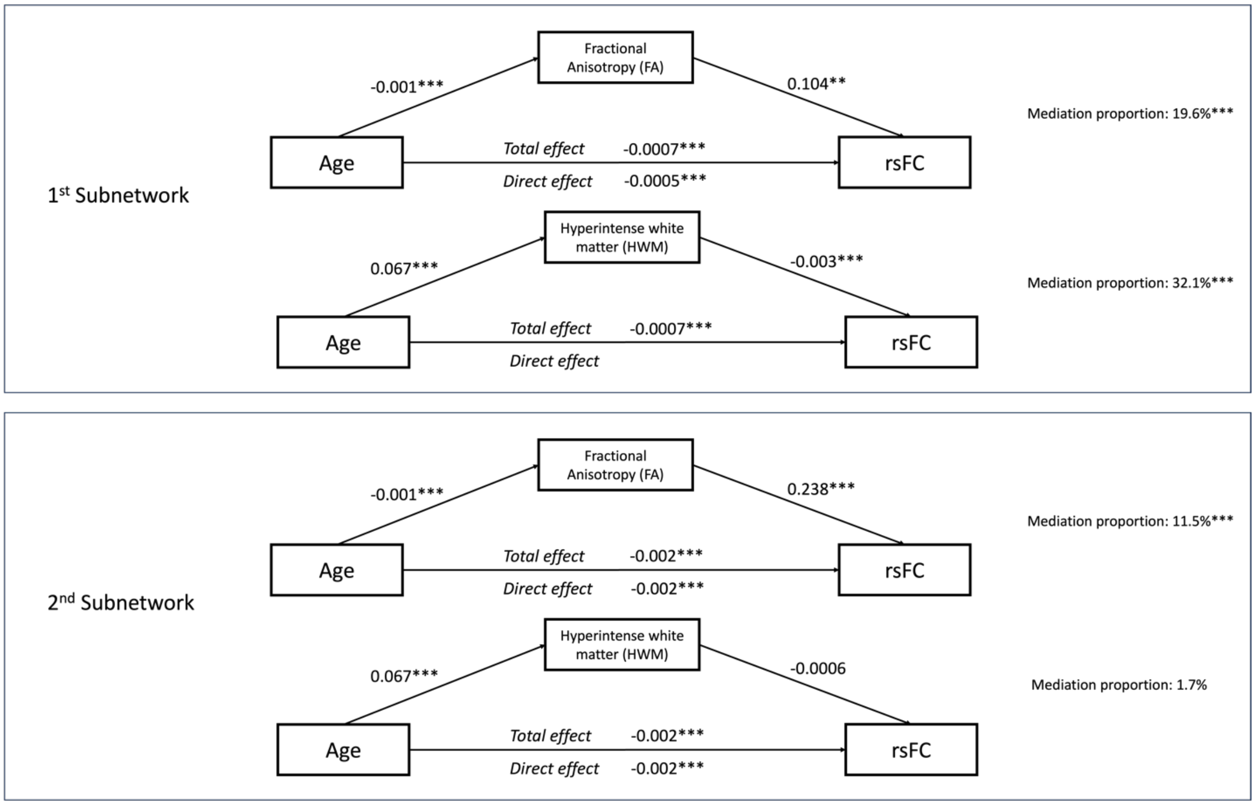

Figure S4. Mediation effect of structural changes on the age-related functional declines. We conducted two path analyses for the data-driven age-related subnetworks, respectively. The top model in each panel examines if average FA mediates the effect of aging on the average rsFC of each subnetwork. The bottom model in each panel studies if the total volume of HWM has a mediating effect on age-related functional declines. All models are adjusted for sex. Asterisks indicate significance levels, with one asterisk (*) representing a 0.05 significance level, two asterisks (**) denoting a 0.01 level, and three asterisks (***) indicating a 0.001 level of significance.

SI.2 Age-related subnetworks by different brain atlases

The primary analysis results using the BNA246 atlas were reproduced using the Schaefer atlas with 200 parcels (Schaefer et al., 2018), the Glasser atlas with 360 parcels (Glasser et al., 2013), and the Power atlas with 264 parcels (Power et al., 2011). The inference matrices with $-\log_{10}(\text{pvalues})$ and t-statistics associated with the age-effect, obtained in the original ROI order of different brain atlases, are displayed in Figure S5.

SI.2.1 Schaefer200

In the UKB cohort, 55 ROIs from the Schaefer200 atlas were identified in the age-related subnetwork, compared to 54 ROIs in the HCP-A cohort, with 36 ROIs overlapping between the two. Of these 36 overlapping ROIs, 24 were from sensorimotor regions, representing 69% of the total sensorimotor areas. Additionally, 3 ROIs were from the dorsal attention network, and 7 from the ventral attention network. Since the Schaefer200 atlas does not include subcortical parcellations, basal ganglia regions were not available for the analysis.

SI.2.2 Glasser360

Using the Glasser360 atlas, 38 ROIs were identified as part of the age-related subnetwork in both the UKB and HCP-A cohorts. Of these, 18 ROIs overlapped between the cohorts, including 9 from the auditory network, 6 from the cingulo-opercular network, and 3 from the somatomotor network. The network mapping of Glasser360 ROIs was derived from a previously published study (Ji et al., 2019). Similar to Schaefer200, Glasser360 does not include subcortical regions, excluding basal ganglia from the analysis.

SI.2.3 Power264

In the Power264 atlas, the age-related subnetwork comprised 63 ROIs in the UKB cohort and 69 ROIs in the HCP-A cohort, with 34 overlapping regions. These ROIs spanned various networks, including auditory, sensorimotor, dorsal/ventral attention, and visual networks. Caudate, putamen, and pallidum were included in the Power264 parcellation, but only bilateral caudate were identified as part of the age-related subnetwork.

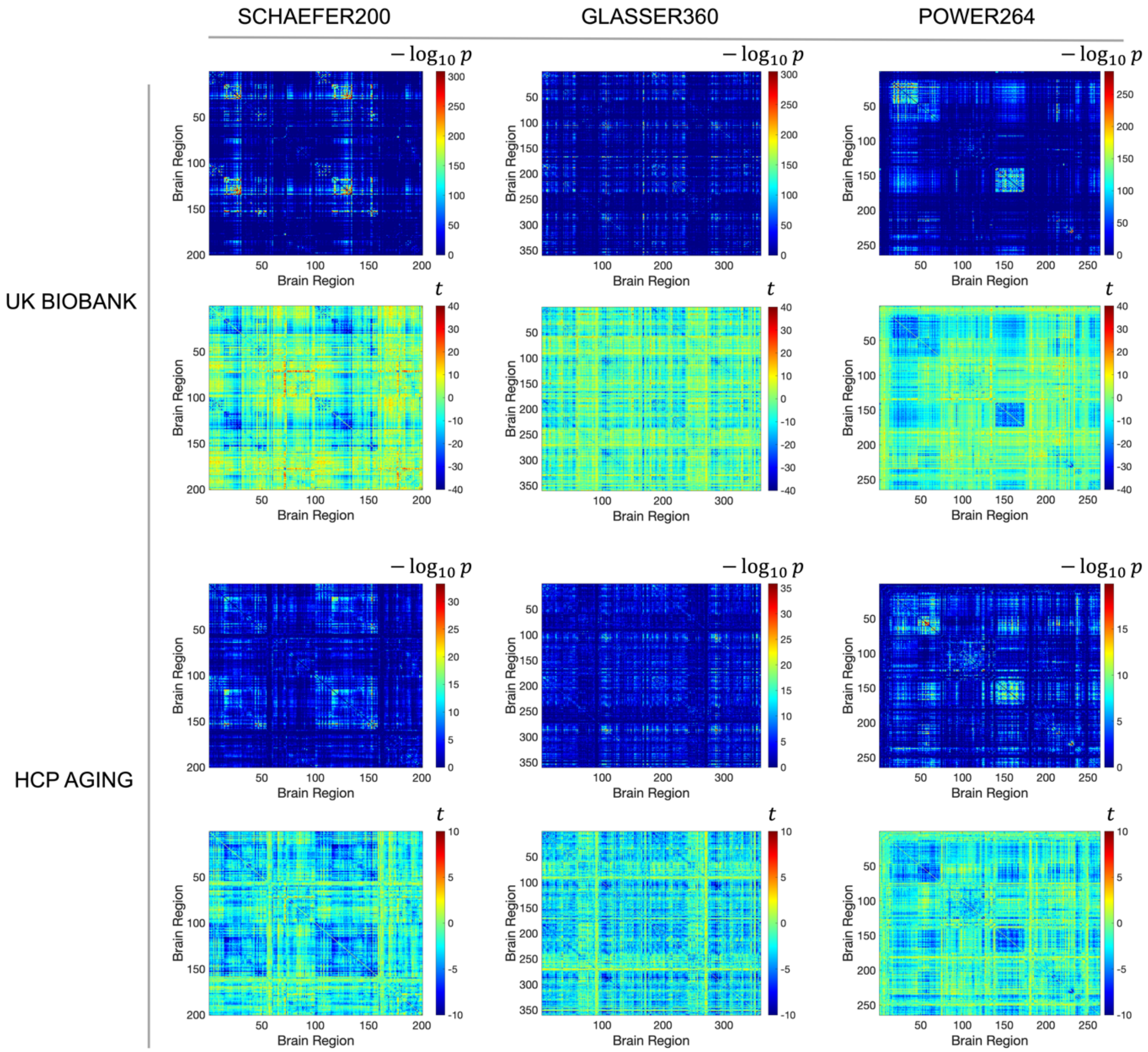

Figure S5. Whole-brain functional connectome derived from various brain atlases, showing age-related differences. The figure displays inference matrices of -log10(p-values) and t-statistics, representing the significance and magnitude of age-related effects on individual rsFC edges, with regions ordered according to the original ROIs from the Schaefer200, Glasser360, and Power264 atlases. In the -log10(p-value) matrices, higher values (depicted in red) indicate greater statistical significance. In the t-statistic matrices, negative values (shown in blue) represent age-related decreases in rsFC, while positive values (shown in red) correspond to age-related increases.

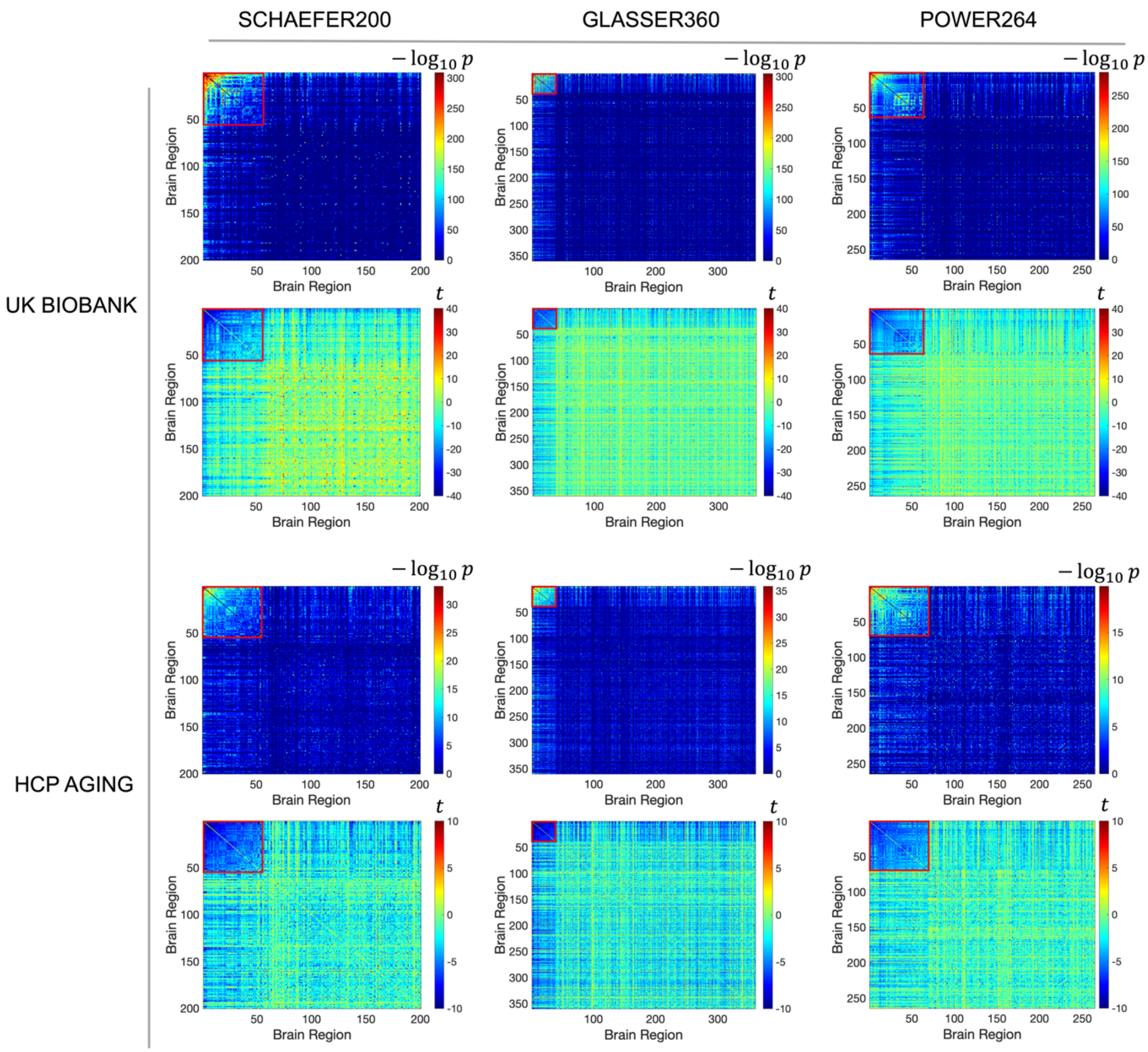

Figure S6. Age-related subnetworks derived from various brain atlases. Age-related subnetworks derived from various brain atlases. The inference matrices of -log10(p-values) and t-statistics, representing the significance and magnitude of age-related effects on individual resting-state functional connectivity (rsFC) edges, were reordered to highlight the extracted subnetworks, outlined by the red box. In the -log10(p-value) matrices, higher values (in red) indicate greater statistical significance. In the t-statistic matrices, negative values (in blue) represent age-related decreases in rsFC, while positive values (in red) reflect age-related increases. The age-related subnetworks show good reproducibility across the UKB and HCP-A cohorts.

Reference

Alfaro-Almagro, F., Jenkinson, M., Bangerter, N. K., Andersson, J. L. R., Griffanti, L., Douaud, G., Sotiropoulos, S. N., Jbabdi, S., Hernandez-Fernandez, M., Vallee, E., Vidaurre, D., Webster, M., McCarthy, P., Rorden, C., Daducci, A., Alexander, D. C., Zhang, H., Dragonu, I., Matthews, P. M., … Smith, S. M. (2018). Image processing and Quality Control for the first 10,000 brain imaging datasets from UK Biobank. *NeuroImage*, *166*, 400–424. https://doi.org/10.1016/j.neuroimage.2017.10.034

Glasser, M. F., Sotiropoulos, S. N., Wilson, J. A., Coalson, T. S., Fischl, B., Andersson, J. L., Xu, J., Jbabdi, S., Webster, M., Polimeni, J. R., Van Essen, D. C., & Jenkinson, M. (2013). The Minimal Preprocessing Pipelines for the Human Connectome Project. *NeuroImage*, *80*, 105–124. https://doi.org/10.1016/j.neuroimage.2013.04.127

Jenkinson, M., Bannister, P., Brady, M., & Smith, S. (2002). Improved Optimization for the Robust and Accurate Linear Registration and Motion Correction of Brain Images. *NeuroImage*, *17*(2), 825–841. https://doi.org/10.1006/nimg.2002.1132

Ji, J. L., Spronk, M., Kulkarni, K., Repovš, G., Anticevic, A., & Cole, M. W. (2019). Mapping the human brain's cortical-subcortical functional network organization. *NeuroImage*, *185*, 35–57. https://doi.org/10.1016/j.neuroimage.2018.10.006

Power, J. D., Cohen, A. L., Nelson, S. M., Wig, G. S., Barnes, K. A., Church, J. A., Vogel, A. C., Laumann, T. O., Miezin, F. M., Schlaggar, B. L., & Petersen, S. E. (2011). Functional network organization of the human brain. *Neuron*, *72*(4), 665–678. https://doi.org/10.1016/j.neuron.2011.09.006

Salimi-Khorshidi, G., Douaud, G., Beckmann, C. F., Glasser, M. F., Griffanti, L., & Smith, S. M. (2014). Automatic denoising of functional MRI data: Combining independent component analysis and hierarchical fusion of classifiers. *NeuroImage*, *90*, 449–468. https://doi.org/10.1016/j.neuroimage.2013.11.046

Schaefer, A., Kong, R., Gordon, E. M., Laumann, T. O., Zuo, X.-N., Holmes, A. J., Eickhoff, S. B., & Yeo, B. T. T. (2018). Local-Global Parcellation of the Human Cerebral Cortex from Intrinsic Functional Connectivity MRI. *Cerebral Cortex (New York, N.Y.: 1991)*, *28*(9), 3095–3114. https://doi.org/10.1093/cercor/bhx179

Smith, S. M., Beckmann, C. F., Andersson, J., Auerbach, E. J., Bijsterbosch, J., Douaud, G., Duff, E., Feinberg, D. A., Griffanti, L., Harms, M. P., Kelly, M., Laumann, T., Miller, K. L., Moeller, S., Petersen, S., Power, J., Salimi-Khorshidi, G., Snyder, A. Z., Vu, A. T., … Glasser, M. F. (2013). Resting-state fMRI in the Human Connectome Project. *NeuroImage*, *80*, 144–168. https://doi.org/10.1016/j.neuroimage.2013.05.039
